# An interdisciplinary forensic approach in a mummified child with evidence of abuse and neglect

**DOI:** 10.1093/fsr/owae049

**Published:** 2024-08-14

**Authors:** Norbert Moravanský, Soňa Masnicová, Petra Švábová, Roman Kuruc, Branislav Gális, Radoslav Beňuš

**Affiliations:** Institute of Forensic Medicine, Faculty of Medicine, Comenius University, Bratislava, Slovak Republic; Institute of Forensic Medical Expertise, Expert Institute, Bratislava, Slovak Republic; Department of Criminalistics and Forensic Sciences, Academy of Police Forces, Bratislava, Slovak Republic; Department of Anthropology, Faculty of Natural Sciences, Comenius University, Bratislava, Slovak Republic; Institute of Forensic Medicine, Faculty of Medicine, Comenius University, Bratislava, Slovak Republic; Department of Forensic Medicine, The Health Care Surveillance Authority, Bratislava, Slovak Republic; Institute of Forensic Medical Expertise, Expert Institute, Bratislava, Slovak Republic; Dental Surgery Clinic, University Hospital Bratislava, Bratislava, Slovak Republic; Department of Oral and Maxillofacial Surgery, Faculty of Medicine, Comenius University and University Hospital Bratislava, Bratislava, Slovak Republic; Institute of Forensic Medical Expertise, Expert Institute, Bratislava, Slovak Republic; Department of Anthropology, Faculty of Natural Sciences, Comenius University, Bratislava, Slovak Republic

**Keywords:** child abuse and neglect, forensic anthropology, forensic pathology, mummified remains, growth arrest lines, inveterate fractures

## Abstract

Mummification of corpses with partial skeletonization is not an uncommon occurrence in daily forensic work. Cooperation between different forensic fields is important in these cases in terms of obtaining the most accurate and forensically relevant results, especially when child abuse and neglect is suspected. In Central Europe, up to 21% of children are exposed to physical and psychological harm, which is mostly perpetrated by family members. This report describes a case of subadult female mummified remains in which interdisciplinary forensic pathology, forensic anthropology, and entomology input was needed to obtain legally relevant results. Entomological analysis of the fly and beetle species present served primarily to estimate the postmortem interval. External examination confirmed advanced postmortem decomposition of the body. The anthropological findings based on radiographs and analysis of selected bones confirmed various antemortem fractures and post-traumatic changes involving the ribs, the distal portion of the humerus, the nasal bones, and the anterior portions of the maxilla and mandible. Furthermore, non-specific findings of growth arrest (Harris) lines in the distal metaphysis of the right tibia indicated growth retardation and, overall, child neglect. The autopsy findings confirmed subdural blood coagulum, part of which formed a clearly moulded plaster mass that had originally been attached to the cranial vault up to the internal lamina. The findings indicated a post-traumatic condition as the underlying cause of death in this child. Interdisciplinary forensic analyses confirmed that the child had been repeatedly exposed to violent assaults throughout her lifetime.

**Key points:**

- If mummified remains with partial skeletonization of a subadult are found, collaboration among forensic experts is required
- In this case, dried subdural blood coagulum indicated post-traumatic, non-spontaneous subdural haemorrhage
- Periosteal reaction present on the facial bones and distal portion of the humerus was a suspicious finding
- Analyses of female subadult remains based on an interdisciplinary approach revealed a syndrome of child abuse and neglect

## Introduction

Child abuse and neglect (CAN) includes both active and passive forms of harm. It also includes non-accidental, conscious (or even unconscious) actions of a parent or another person towards a child that are socially unacceptable, have a negative impact on the child’s physical, sexual, social, and emotional development, and may even have a fatal outcome [1]. A survey of 5 230 Slovakian children aged 15–16 years by Slovak Hope for Children (SLONAD) in 1999 found that almost a quarter of these children had experienced physical assaults, including hitting, kicking, and cutting. More than 12% of the children were victims of sexual abuse, and the perpetrator was someone close to them or someone they knew. The most common forms of domestic violence against children include repeated physical and psychological abuse, verbal abuse, and threats. Two-thirds of those affected are children under the age of 3 years [2]. According to police statistics, there were between ~50 and ~100 cases of CAN per year in Slovakia between 2012 and 2023 (Table 1), 25% to ~46% of which were committed against children under the age of 6 years [3]. It should be noted that in 2020 and 2021, during the COVID-19 pandemic, there was a sharp increase in crimes against minors, with 45.5% of 99 reported cases involving children under 6 years of age. A similar increase in cases of CAN has been noted in other countries, for example, in the state of Texas in the USA [4].

**Table 1:** Overview of offences under Section 208 of the Battering a Close Person and a Person Entrusted into One’s Care of the Criminal Code (Act 300/2005 Coll. of 20 May 2005 Criminal Code) in the Slovak Republic in the years 2012–2023 [3].

Year^a^	Crimes against minors (*n*)	Crimes against children under 6 years of age (*n*)	Percentage^b^
2012	52	13	25.0
2013	53	21	39.6
2014	55	21	38.2
2015	56	22	39.3
2016	96	23	24.0
2017	86	30	34.9
2018	72	19	26.4
2019	83	26	31.3
2020	99	45	45.5
2021	92	31	33.7
2022	71	23	32.4
2023	77	25	32.5

^a^Data collected for the period 1 January 2012 to 31 August 2023.

^b^Data collected for the period 1 January 2012 to 31 August 2023.

Mummification of bodies with partial skeletonization is not an uncommon occurrence in everyday forensic practice. In such cases, interdisciplinary collaboration and use of multiple identifiers can lead to positive identification [5–7]. Collaboration between forensic anthropologists in institutions, forensic pathologists, and the police could lead to a better understanding of the usefulness of forensic anthropology in forensic investigations [8]. In Slovakia, most cases involving skeletal remains are initially handled by forensic pathologists and/or anatomists, after which anthropologists might be involved. Reports in the literature suggest that very few countries engage physical anthropologists for skeletal evaluation [7, 9]. Anthropological analysis complements the pathological findings, especially in cases of skeletal trauma. Collaboration is essential in these criminal cases, and both pathological and anthropological findings are considered a relevant component of the final forensic report. This report describes a case in which a body in an advanced state of decomposition was confirmed to be a case of CAN by interdisciplinary collaboration between experts in forensic anthropology, forensic pathology, and entomology.

## Case report

**Figure 1:**
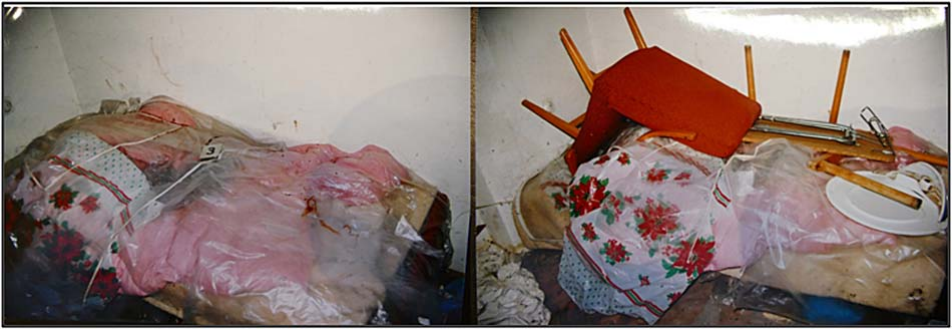
Investigation of the crime scene with a focus on the bed.

**Figure 2:**
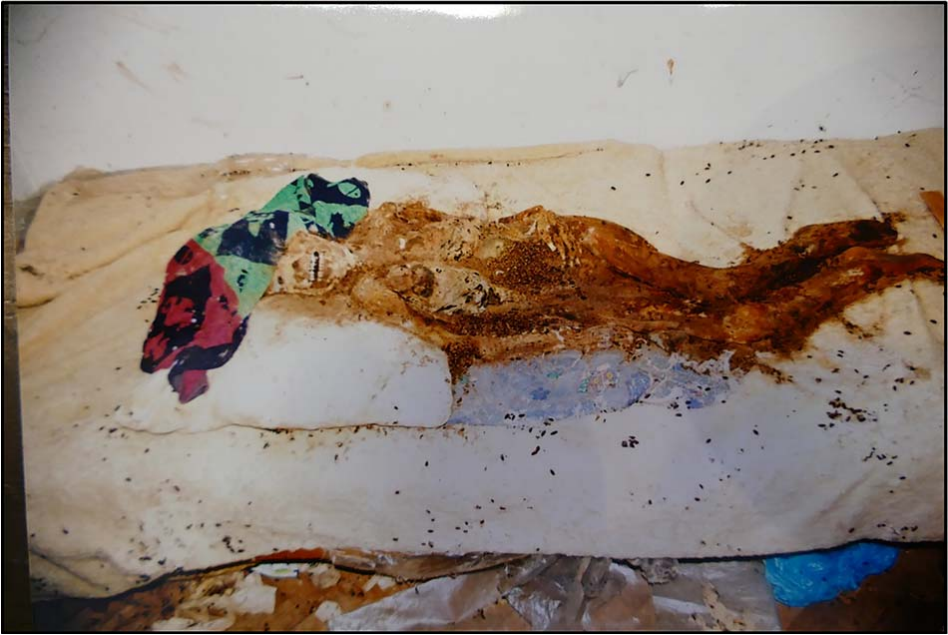
Mummified remains with insect pupae found on a blanket.

In September 2012, mummified human remains and various objects were found on a bed in a locked and barricaded room in the home of the victim’s family in Bratislava, the capital of Slovakia **(**Figure 1**)**. The body was lying on its back on a white sheepskin blanket with a pillow made of cloth under the head and torso. Insect pupae and insect damage were widely distributed over the remains (Figure 2)**.** According to the stepfather’s statement, he had beaten his stepdaughter, whereupon she started to tremble and lost consciousness. He then put her to bed. On the next day, the stepfather and his wife (the child’s biological mother) found the child to be deceased. After a few days, when decomposition had probably started and insects were starting to accumulate on the child’s body, he covered her with a bedspread and other items in the room. He then locked the room, left the window open, covered the door with a blanket, and pushed a wardrobe against the door. Thereafter, no one entered the room for 3 years. When the neighbours enquired about the child, the mother said that she had left her daughter with her sister because her husband could not accept her.

### External and internal examination

**Figure 3:**
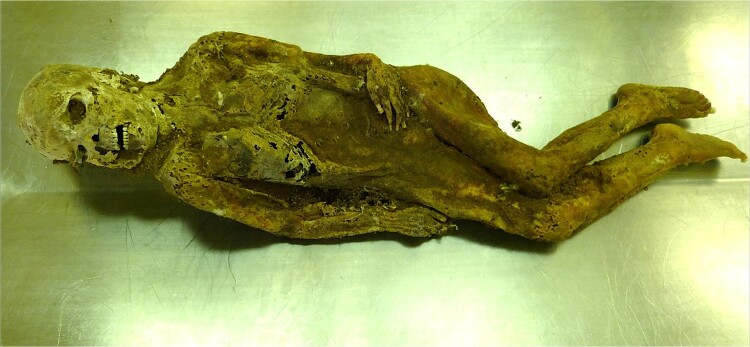
A mummified corpse with partial skeletonization in the autopsy room.

External examination of the unclothed body revealed an advanced state of decomposition with mummification and skeletonization of the skull and its facial components. Several postmortem skin defects caused by insect larvae and desiccation were also found **(**Figure 3**).** Experts at the Zoological Institute of the Slovak Academy of Sciences found remains of *Calliphora vicina* and larval skins, imagos, and live larvae of *Dermestes lardarius*. A demarcated darkish-brown to black area was found on the back of the head. No obvious evidence of violent assault was found on the preserved skin of the neck, chest, abdomen, lower limbs, and back, including the fingers and nails.

**Figure 4:**
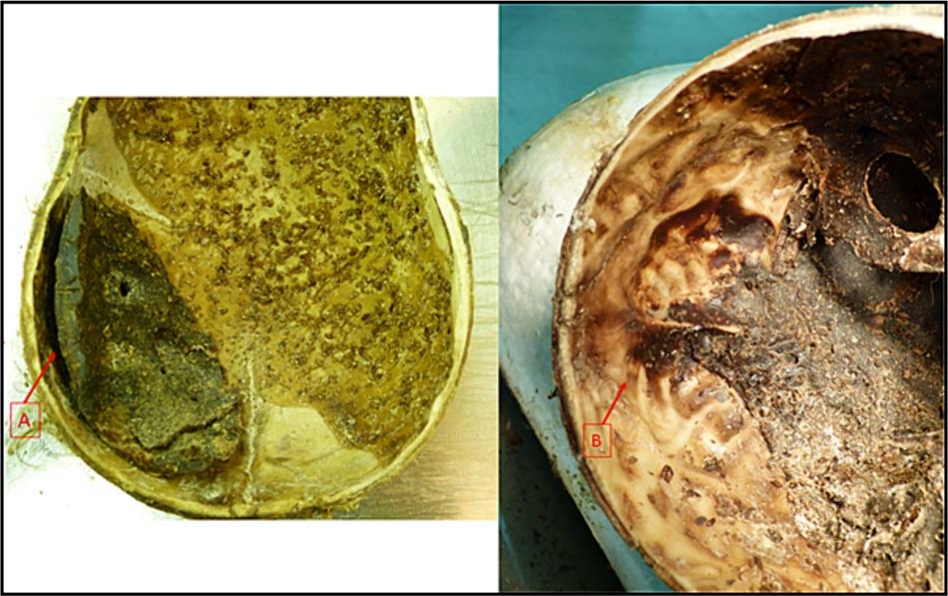
Cranial cavity of investigated mummified remains. Presence of dried blood coagulum in the cranial cavity (A) and dark discoloration of the lamina interna (B).

At autopsy, dried blood coagulum was found in the subdural cranial space in the area from the occipital bone to the temporal region on the right (Figure 4A). Darkish-brown and black discolouration of the intact lamina interna of the cranial vault and base was noted (Figure 4B). The internal organs had completely disintegrated, except for the remains of the intestinal tract in the abdominal cavity. No obvious traumatic changes were found in the preserved parts of the internal lining of the thorax and abdominal wall.

### Anthropological and radiological findings

**Figure 5:**
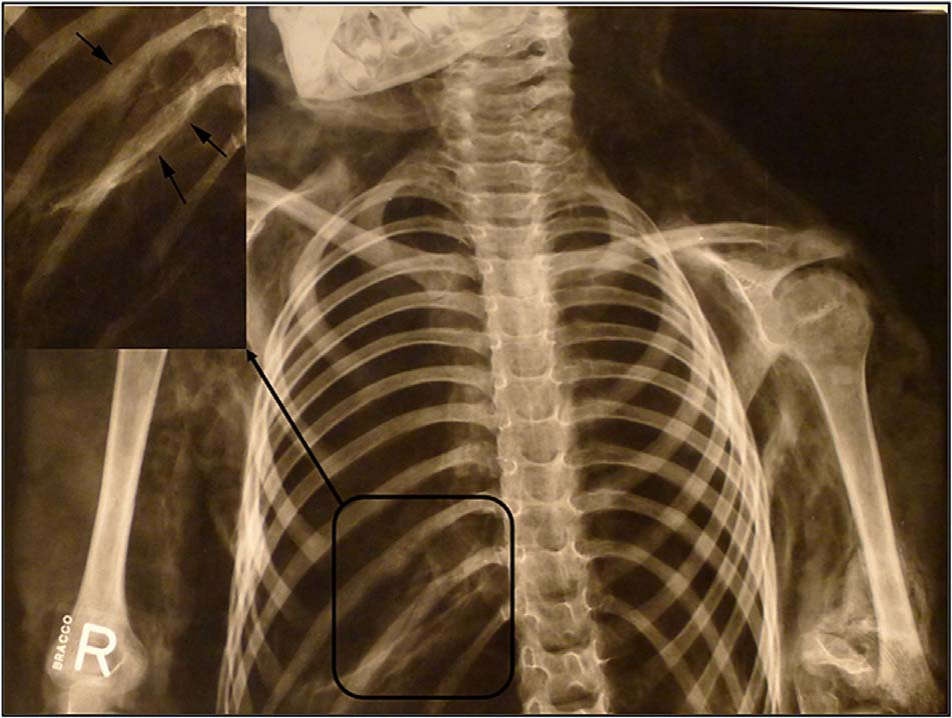
Healed fractures of the 10th and 11th ribs on the right side of the spine.

**Figure 6:**
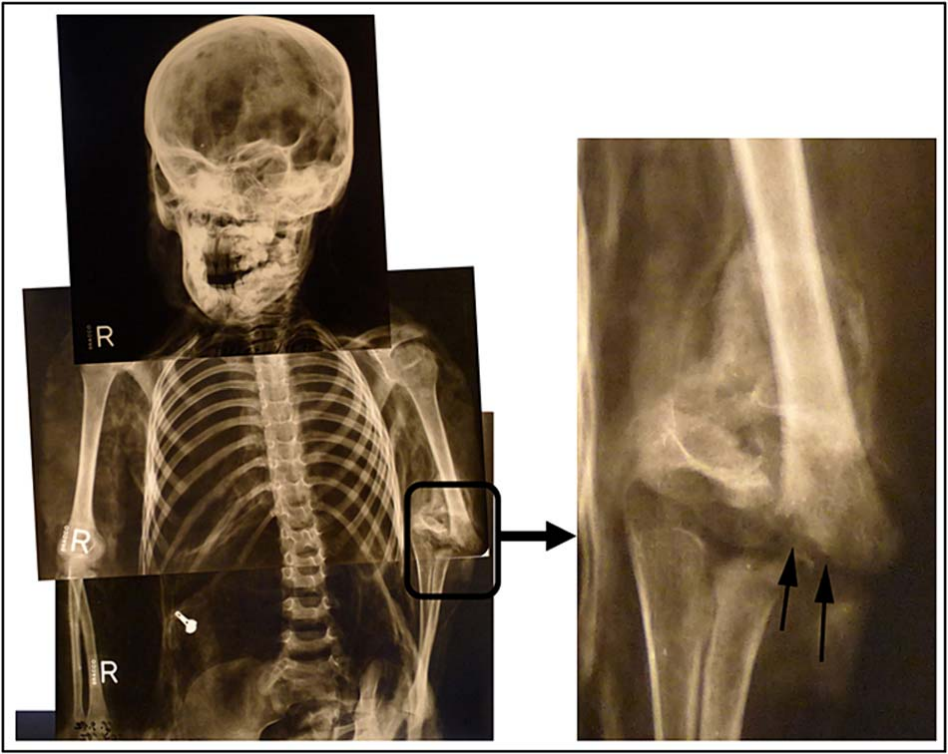
Untreated fracture of the medial epicondyle of the left humerus.

**Figure 7:**
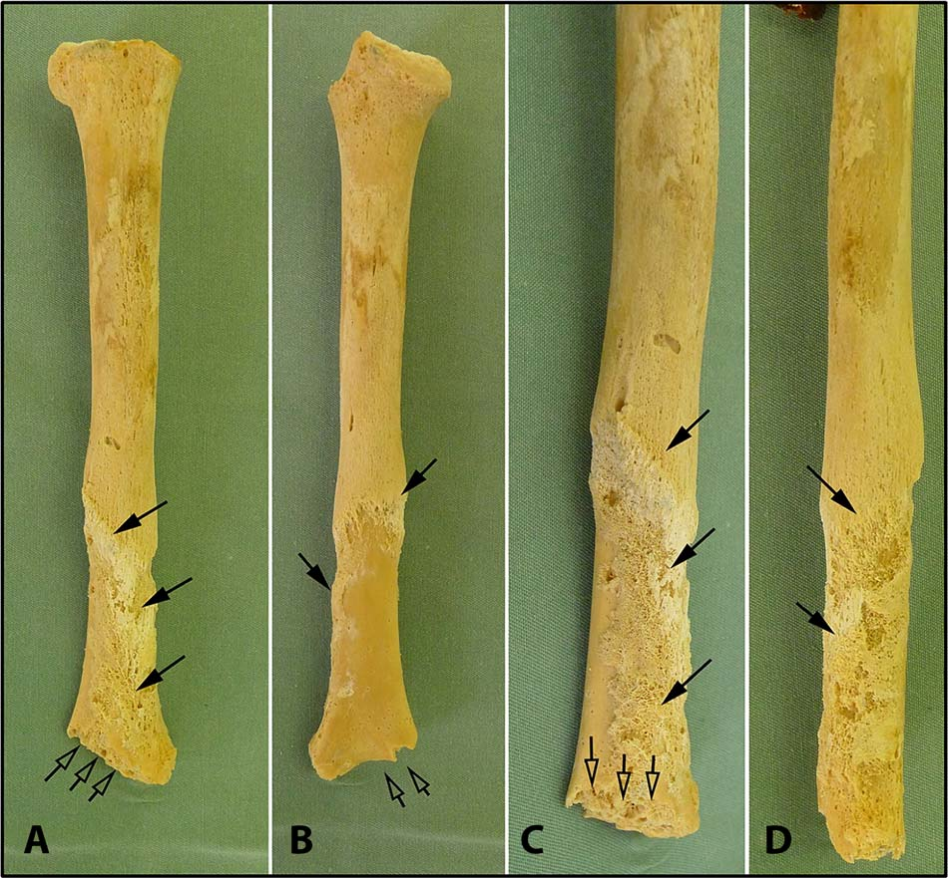
Diaphysis of the left humerus. (A) Anterior view. (B) Posterior view. (C) Detailed anterior view of the distal portion. (D) Detailed lateral view of the distal portion. Full arrows show the periosteal reaction. Empty arrows show the fracture of the medial epicondyle.

**Figure 8:**
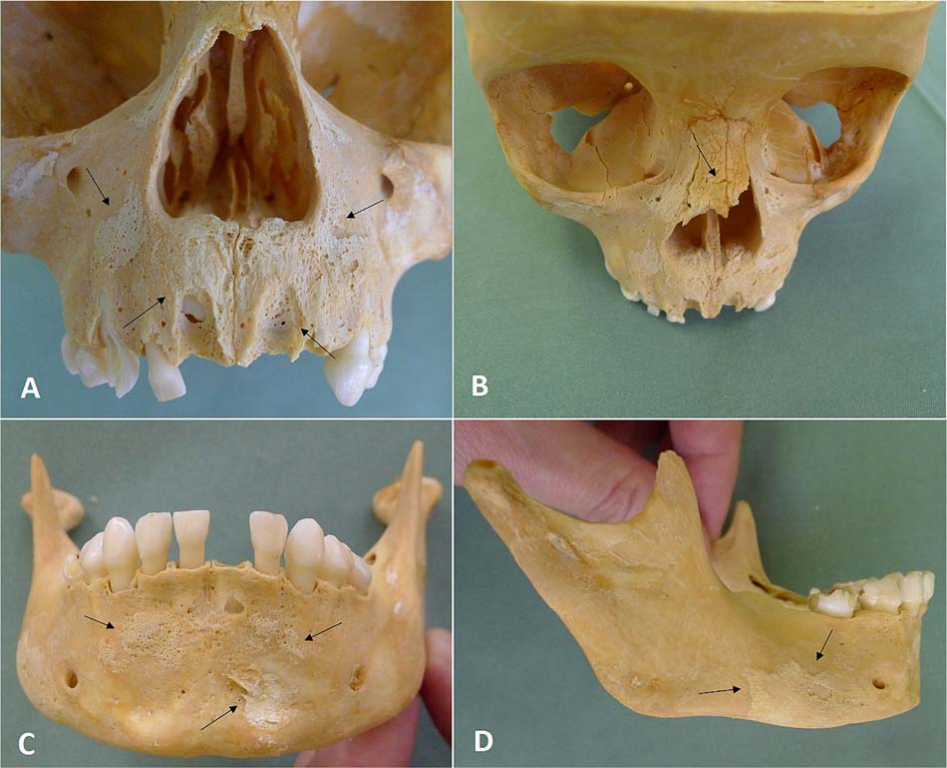
Fractures and periosteal reaction on facial bones. Periosteal reaction on the maxilla (A), fracture of the nasal bone (B), periosteal reaction on the mandible in an anterior view (C) and lateral view (D).

**Figure 9:**
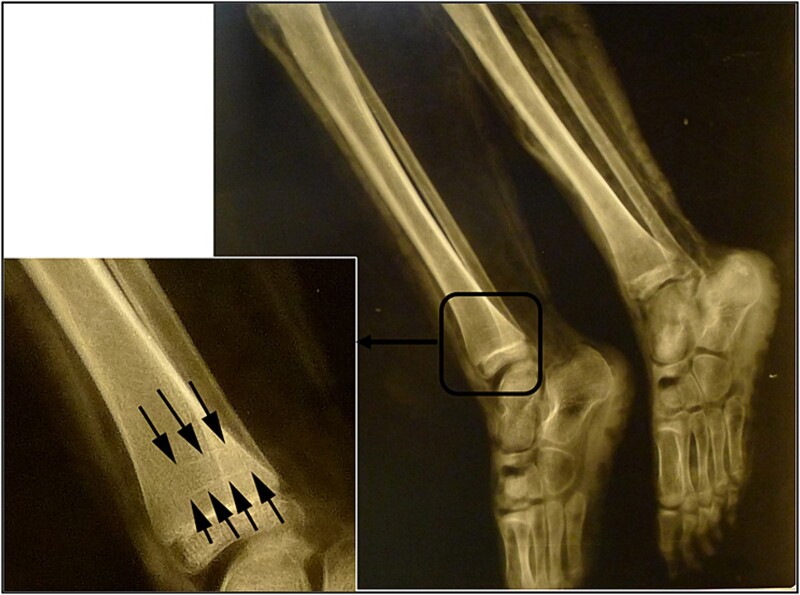
Radiograph showing the Harris lines on the distal metaphysis of the right tibia.

Radiological examination revealed integrity of the cervical, thoracic, and lumbar spine and a congenital anomaly of the second and third cervical vertebrae. Antemortem posterior fractures of the 10th and 11th ribs were confirmed (Figure 5). Furthermore, the left elbow was noted to be in a non-anatomical position, and both radiography and osteological analysis confirmed a fracture of the left medial epicondyle of the humerus and an active periosteal reaction on the lower third to half of the left humerus (Figures 6 and 7). A fracture without signs of healing was observed in the distal third of the nasal bone (Figure 8B). A periosteal reaction was present in the alveolar part of the maxilla as well as in the mentum and on the right side of the mandibular body **(**Figure 8A, C, D). Harris lines were present on the right tibia (Figure 9)**.** No traumatic changes were observed in the pelvis or lower limbs.

### Evaluation of the biological profile and DNA analysis

The mummified remains were 114.5 cm in height with a weight of 4 700 g. On analysis of the remains, the age at death was estimated to be 5–6 years based on the formation and eruption of the dentition and skeletal characteristics [10, 11]. The biological sex of the child was estimated to be female based on the results of DNA analysis and external examination of the mummified genitalia. DNA analysis confirmed that the child was biologically related to the mother but not to the stepfather. These findings confirmed the child’s identity.

### Microscopic findings

Microscopic histological examination of the dried blood coagulum revealed advanced putrefactive changes and structures very similar to brain tissue. Serological and haematological examination confirmed that a sample from the coagulum was positive for the presence of blood. No toxicologically significant substances (including drugs and narcotics or their metabolites) were detected.

## Discussion

### Estimate of postmortem interval based on entomological findings

The remains of imago-stage *C. vicina*, a widespread urban fly species that is attracted to fresh dead bodies and active in the spring and autumn months [12, 13], indicated that the death probably occurred in spring or autumn. If the child had died earlier (i.e., in winter), species of the genus *Lucilia* would have been attracted to the body. The absence of the genera *Lucilia, Phormia,* and *Pollenia* indicated that the death had not occurred in summer. Larvae, imago, and live larvae of *D. lardarius* were found on the body at the same time. These necrophagous beetles have a longer larval period than flies, making them useful in the late decomposition stage for estimating the minimum postmortem interval [14]. They infest bodies that have been dead for 3–6 months and produce only one generation per year, so it can be assumed that death had occurred at least 2 years earlier.

### Determination of cause of death and injuries by forensic pathologists

The mummified haematoma in the subdural space of the skull represented a post-traumatic haemorrhage into the cranial cavity and was the immediate cause of the child’s death. The mode of injury was blunt force trauma to the head. According to the autopsy findings, an external mechanical force of a blunt nature acting in a vectorial direction caused the subdural haemorrhage and haematoma. The majority (75%–80%) of abused children die from head injuries [15]. A spontaneous fall from a standing height resulting in impact to the head and subdural haematoma would be very rare and not result in such a severe injury in a child under the age of 6.5 years during normal activity. External force trauma is more likely in a victim of abuse [16]. Paediatric abusive head trauma usually involves injury to the intracranial contents or the skull of an infant or child under 5 years of age by violent shaking or blunt impact. This condition is also known as shaken baby syndrome. Children diagnosed with paediatric abusive head trauma have a 5-fold higher risk of death than children with an accidental head injury [17, 18]. According to the stepfather’s statement, the child has protested about being made to sit on a potty and hit her head after falling off the potty onto the floor. No other mechanisms were found at autopsy to explain the child’s death.

### Analysis of post-traumatic changes by forensic anthropologists

The timing of trauma according to age group is important in analysis of fractures because it can provide reliable evidence of physical abuse [19]. Detection of metaphyseal and rib fractures in the younger age groups is considered highly specific for abuse and is strongly correlated with maltreatment in young children. Therefore, non-accidental trauma should be considered when such fractures are found [20, 21].

The finding of an active periosteal reaction on the lower third to half of the left humerus indicated that the bone had been subjected to excessive stress during the child’s lifetime by twisting of the upper limb about its longitudinal axis, most likely by the actions of another person, resulting in closed or open fracture. Such findings are common in abused children [22, 23]. According to the stepfather’s statement, he had held her hand while she was bathing, whereupon she slipped, fell, and dislocated her elbow. He wrapped a cloth around the elbow and bandaged it but did not consult a doctor. Depending on the type of periosteal reaction, it is possible to pinpoint its onset to about 4 weeks before death [21]. A fracture with periosteal injury typically causes severe pain, especially in children. Without adequate treatment, after the pain has temporarily subsided, there may be a flare-up of severe pain because of inadequate fixation of the limb at the site of the fracture and formation of purulent inflammation at the fracture site, as reported by the stepfather in this case. Furthermore, within a few days of the fracture, a fistula with purulent inflammation could form on the surface of the elbow joint with possible exposure of the skin covering the elbow and appearance of a bone fragment.

Rib fractures in children are strongly associated with non-accidental trauma and child abuse, particularly posterior rib fractures [21, 24, 25]. In the present case, the ante-mortem posterior fractures of the 10th and 11th ribs that were in an advanced stage of healing (and could have occurred in the order of several weeks to months before death [26]) indicated blunt force trauma to the chest from the outside, more likely from the side or posterior right direction.

The periosteal reaction in the anterior alveolar part of the maxilla, in the mentum, and on the right side of the mandibular body indicated blunt force trauma from an external source, which may have been a single blow or repeated blows, possibly a slap or punch from another person or repeated falls on the anterior facial area. According to witnesses, the child was frequently slapped on the face by her mother.

Growth arrest lines (also known as growth resumption lines and Harris or Park lines) in the distal metaphysis of the right tibia in children are usually caused by malnutrition, disease, or trauma, but may also be the result of non-specific stress during bone growth, alternating periods of normal growth and growth spurts, or delayed development [27]. In the present case, this non-specific finding was the only indicator of growth retardation, which could also be related to specific forms of maltreatment associated with the syndrome of CAN. It could be an important finding in terms of the overall assessment of the possible care of the child or a consideration of the changes in the injuries.

## Conclusions

To our knowledge, the present case is the first of its type in Slovakia. This report on the mummified remains of a female child emphasizes the importance of collaboration between forensic anthropologists and pathologists in criminal cases. Use of forensic anthropologists is particularly important in cases of suspicious, non-accidental skeletal injuries. The expert conclusions in this case indicate that the child was repeatedly exposed to violent physical assaults throughout her lifetime. The syndrome of CAN interferes with a child’s physical, mental, and social development and may even culminate in death. A comprehensive approach that includes prosecution of perpetrators, protection of victims, and prevention of this phenomenon is required. In this case of subadult female mummified remains, the impact of an external force of a blunt nature caused subdural haemorrhage and haematoma. Further analysis by a forensic anthropologist confirmed post-traumatic skeletal changes, namely periosteal reaction on the facial bones, humerus, and ribs. The entomological findings confirmed that death occurred at least 24 months before the body was found, which is consistent with the stepfather’s statement. The forensic expert pointed out trauma-related changes in this case that required medical treatment. However, according to the investigators, the child had not been taken to the paediatric outpatient clinic. The case had obvious hallmarks of failure to provide assistance to a child by one or more adults who were or could be perpetrators of or witnesses to the repeated harm inflicted on this child.
